# Dietary Fiber Pectin Directly Blocks Toll-Like Receptor 2–1 and Prevents Doxorubicin-Induced Ileitis

**DOI:** 10.3389/fimmu.2018.00383

**Published:** 2018-03-01

**Authors:** Neha M. Sahasrabudhe, Martin Beukema, Lingmin Tian, Berit Troost, Jan Scholte, Erik Bruininx, Geert Bruggeman, Marco van den Berg, Anton Scheurink, Henk A. Schols, Marijke M. Faas, Paul de Vos

**Affiliations:** ^1^Immunoendocrinology, Division of Medical Biology, Department of Pathology and Medical Biology, University Medical Center Groningen, Groningen, Netherlands; ^2^Laboratory of Food Chemistry, Wageningen University, Wageningen, Netherlands; ^3^Faculty of Mathematics and Natural Sciences, Neuroendocrinology, Groningen Institute for Evolutionary Life Sciences, Groningen, Netherlands; ^4^Agrifirm Innovation Center, Apeldoorn, Netherlands; ^5^Animal Nutrition Group, Wageningen University, Wageningen, Netherlands; ^6^Nuscience Group Headquarters, Ghent, Belgium; ^7^DSM Biotechnology Center, Delft, Netherlands; ^8^Department of Obstetrics and Gynaecology, University of Groningen, University Medical Center Groningen, Groningen, Netherlands

**Keywords:** dietary fiber, toll-like receptor 2, pectin, ileitis, degree of methyl esterification

## Abstract

Dietary carbohydrate fibers are known to prevent immunological diseases common in Western countries such as allergy and asthma but the underlying mechanisms are largely unknown. Until now beneficial effects of dietary fibers are mainly attributed to fermentation products of the fibers such as anti-inflammatory short-chain fatty acids (SCFAs). Here, we found and present a new mechanism by which dietary fibers can be anti-inflammatory: a commonly consumed fiber, pectin, blocks innate immune receptors. We show that pectin binds and inhibits, toll-like receptor 2 (TLR2) and specifically inhibits the proinflammatory TLR2–TLR1 pathway while the tolerogenic TLR2–TLR6 pathway remains unaltered. This effect is most pronounced with pectins having a low degree of methyl esterification (DM). Low-DM pectin interacts with TLR2 through electrostatic forces between non-esterified galacturonic acids on the pectin and positive charges on the TLR2 ectodomain, as confirmed by testing pectin binding on mutated TLR2. The anti-inflammatory effect of low-DM pectins was first studied in human dendritic cells and mouse macrophages *in vitro* and was subsequently tested *in vivo* in TLR2-dependent ileitis in a mouse model. In these mice, ileitis was prevented by pectin administration. Protective effects were shown to be TLR2–TLR1 dependent and independent of the SCFAs produced by the gut microbiota. These data suggest that low-DM pectins as a source of dietary fiber can reduce inflammation through direct interaction with TLR2–TLR1 receptors.

## Introduction

Lower intake of dietary fiber by Westerners (1) coincides with a higher prevalence of certain diseases, such as diabetes (2), obesity (3), asthma (4), allergies (5), inflammatory bowel disease (6), and colorectal cancer (7). A number of these typical Western diseases, including asthma, allergies, and inflammatory bowel diseases have an immunological basis (8). Lower consumption of dietary fibers has been proposed as the leading cause for the rise in immunological disorders in western societies (8–10). However, the underlying mechanisms that link dietary fiber consumption and immunity are largely unknown. Short-chain fatty acids (SCFAs) are one of the mediators (11). Dietary fibers may serve as fermentation substrate for gut microbiota, which generate SCFAs that in turn attenuate inflammation by enhancing numbers of immune regulatory T_reg_ cells in the intestine (12). However, beneficial effects of dietary fibers independent of SCFAs have also been reported (13, 14), wherein direct immunomodulatory effects of dietary fibers on immune cells were proposed (15). More knowledge on mechanisms of beneficial effects of dietary fibers is required for a better insight in their potential application for health promoting effects.

Apart from a need for mechanistic insight, there is also a scientific and societal need to determine which dietary fibers are beneficial for immunity (16). Studies on the relation between dietary fiber intake and Western diseases make no distinction in types of fibers (4, 5), while it has been shown *in vitro* that only specific fibers contribute to immunity (15, 17). Also, health advisory groups only recommend on quantitative intake and not on consumption of specific types of fibers (16). Previous studies from us and others have shown that immunomodulation capacities of dietary fibers can differ even within one type of fiber. It was demonstrated that chemical differences such as variations in side chains and in chain length determine immune effects (15, 17). Knowledge on the role of chemical structure on immunity such as effect of branching, and chain length of dietary fibers are therefore essential to understand and predict efficacy of dietary fibers.

Pectin is one such widely consumed dietary fiber having immunomodulatory effects. Pectin is the structural component of plant cell walls and is abundantly present in fruits. Pectins have α(1-4)-linked galacturonic acid backbone and can differ in degree of methyl esterification (DM). Dietary effects of pectin have been shown to be effective against endotoxin-induced inflammations (18–21) allergic airway inflammations (22), colitis (23), pancreatitis (24), and in supporting colonic anastomosis healing (25) in various animal models. Although anti-inflammatory effects of pectin are recognized, only a few studies have addressed mechanistic differences of pectins with different DM values (18, 24, 25) and none of the studies addressed true binding or identification of specific immune receptor responsible for the anti-inflammatory effects.

We hypothesized that the chemistry dependent effects of pectins may be caused by different binding capacities to interact with the so-called pattern recognition receptors (PRRs), as it has been demonstrated that some dietary fibers can have direct effects on immune cells by directly stimulating these innate immune sensors (15, 17, 26). Toll-like receptors (TLRs) are the best characterized PRRs expressed in the intestine (27–29) and have been shown to be involved in recognition of dietary fibers (17). Here, we studied the relation between DM value of pectins and the capacity to activate or inhibit TLRs as a possible mechanism by which pectins may have versatile immunomodulatory properties. To determine potential structure–function relationships we compared different citrus pectins having a range of DM levels. These pectins, except for a DM variation, have a relatively similar chemical structure allowing sound comparison of DM effects on TLR activation or inhibition. We investigated the effect on extracellular TLRs; TLR2, TLR4, and TLR5. The effects on TLR2 were also studied for the combined effects with the two heterodimers TLR2 forms with TLR1 and TLR6. This was done because TLR2 is unique in its ability to form heterodimers with other membrane receptors such as TLR1 or TLR6 to stimulate different activation pathways (30).

Here, we show that pectin with a low DM has predominantly TLR2 inhibiting effects similar to that of TLR2 antibodies while higher DM pectins are ineffective. Low-DM pectins interact with the TLR2–TLR1 heterodimer, which was confirmed by using an approach of ELISA containing tagged TLR2 and application of mutants of TLR2 in which potential binding sites were modified. To demonstrate principle applicability of the acquired structural insights, we tested the pectin efficacy in preventing TLR2-dependent ileitis in a mice model. Our work provides to our opinion novel insights in structure–function effector relationships of dietary fibers and enables us to rationalize contradictory findings on immune efficacy of dietary fibers. Also, our work provides novel insight in mechanisms of action of dietary fibers and can assist in generating more effective functional food formulations for promoting immune health and eventually contribute to a strategy for prevention of Western diseases.

## Materials and Methods

### Pectin Samples

Commercially extracted lemon pectins with different degree of methyl esterification (DM) (DM7, DM22, DM45, DM60, and DM75) were obtained from CP Kelco (Lille Skensved, Denmark). Endotoxin levels in pectin samples were confirmed with endotoxin detection kit (Thermo Scientific, Sunnyvale, CA, USA), and endotoxin levels were below detection level of 0.1 ng/ml.

### Characterization of Pectins

High performance size exclusion liquid chromatography was performed on an Ultimate 3000 HPLC system (Thermo Scientific, Sunnyvale, CA, USA). A Super AW-L guard column (4.6 mm i.d. × 35 mm, Tosoh Bioscience, Tokyo, Japan) and three TSK-gel columns (6 mm i.d. × 150 mm) connected in series (4000, 3000, and 2500 SuperAW Tosoh Bioscience, Tokyo, Japan) were used for analysis. A sample of 20 µl (2.5 mg/ml in 10 mM sodium acetate buffer, pH 5.0) was injected and eluted with 0.2 M sodium nitrate at a flow rate of 0.6 ml/min at 55°C. The HPLC system was controlled by Chromeleon version 7. Detection was achieved with a refractive index detector Shodex R101 (Showa Denko, Japan). The molecular mass of polysaccharides was calculated using a calibration curve using pullulan standards (Sigma, St. Louis, MO, USA).

The DM of polysaccharides were determined by adding 0.8 mL of 0.4 N sodium hydroxide in 2-propanol/water (50/50 v/v) to 10 mg pectin for 4 h and analyzing the acetic acid and methanol released by HPLC (31). The DM was calculated as moles of acetic acid or methanol per 100 mol of UA.

The constituent monosaccharide content and composition was determined by gas–liquid chromatography (32). Samples were pre-hydrolyzed with 72% (w/w) H_2_SO_4_ for 1 h at 30°C followed by hydrolysis with 1 M H_2_SO_4_ for 3 h at 100°C. The monosaccharides released were derivatized into their alditol acetates and determined by gas chromatography (Focus-GC, Thermo Scientific, MA, USA) using inositol as an internal standard. Uronic acid content was determined as anhydro-uronic acid content using an automated colorimetric m-hydroxydiphenyl assay (33) including 0.3% (w/w) tetraborate in the sulfuric acid.

### Cell Lines

To study the interaction of pectin with TLRs, we used the HEK-Blue™ TLR cell based assays from InvivoGen, Toulouse, France. These HEK-Blue™ cells express the soluble embryonic alkaline phosphatase (SEAP) gene, which can be quantified using Quanti-Blue (InvivoGen, Toulouse, France). The SEAP gene is under the control of the NF-κB/AP-1 responsive promoter. These HEK-Blue™ cells also co-express single TLR genes. Upon activation with TLR specific agonists, NF-κB is activated leading to SEAP expression. We used HEK-Blue™ hTLR4, hTLR5 cells (InvivoGen, Toulouse, France), and HEK-Blue™ Null1 TLR2 (developed by us) for studying the interaction of TLRs and pectins. HEK-Blue™ Null1 (InvivoGen, Toulouse, France) is the parental cell line for HEK-Blue™ TLR cells, expressing the SEAP gene, but not any TLR expression construct (34). Cell lines, antibiotics, and concentration of agonists used to activate TLR signaling are shown in Table 1. Mouse macrophage cells RAW264.7 (ATCC, VA, USA) were used to determine TLR2 inhibition using pectin.

**Table 1:** The toll-like receptors (TLR) reporter cell lines, their agonists, and selection antibiotics.

Name of the cell line (InvivoGen)	Agonist (InvivoGen)	Selection antibiotics (InvivoGen)
HEK-Blue™ hTLR4	10 ng/ml LPS	HEK-Blue™ Selection (1X)
HEK-Blue™ hTLR5	10 ng/ml Flagellin	Blasticidin (30 µg/ml) Zeocin (100 µg/ml)
HEK-Blue™ Null1 TLR2	100 ng/ml Pam3CSK4	Zeocin (100 µg/ml) Hygromycin B (150 µg/ml)
HEK-Blue™ hTLR2 CD14	10^7^ cells/ml Heat killed *Listeria monocytogenes*	HEK-Blue™ Selection (1X)

HEK cells were cultured in DMEM culture media (Lonza, Basel, Switzerland), and RAW264.7 were cultured in RPMI culture media (Lonza, Basel, Switzerland) with 10% de-complemented fetal calf serum, 50 U/ml penicillin (Sigma, St. Louis, MO, USA), 50 µg/ml streptomycin (Sigma, St. Louis, MO, USA), and 100 µg/ml normocin (InvivoGen, Toulouse, France).

### TLR2 Ectodomain Expression

RNA was extracted from HEK-Blue™ hTLR2 (InvivoGen, Toulouse, France) cells using RNeasy^®^ Plus Mini kit (Qiagen, Venlo, The Netherlands). cDNA was synthesized using OligodT primers (Life Technologies, Carlsbad, CA, USA), dNTP mix (Life Technologies, Carlsbad, CA, USA), and Superscript™III Reverse Transcriptase (Life Technologies, Carlsbad, CA, USA). The first 586 codons of TLR2 were amplified from HEK-Blue™ hTLR2 cDNA using forward primer 5′ GCGCACCGGTATGCCACATACTTTGTGGATGG 3′ and reverse primer 5′ GCGCGGATCCGTGACATTCCGACACCGAGAG 3′ with Pfu DNA polymerase (Thermo Scientific, Waltham, MA, USA). Primers were flanked by a 5′ GC doublet for improved restriction enzyme digestion. Underlined letters represent AgeI and BamHI restriction sites for forward and reverse primers, respectively. Fast digest restriction enzymes (Thermo Scientific, Waltham, MA, USA) were used to digest the PCR product and pSELECT-CHA-blasti (InvivoGen, Toulouse, France) to create sticky ends. After ligation using T4 DNA Ligase (Thermo Scientific, Waltham, MA, USA), the mixture was used to transform One Shot TOP10 Chemically Competent *E. coli* (Life Technologies, Carlsbad, CA, USA). Transformed *E. coli* were selected on Blasticidin agar media (InvivoGen, Toulouse, France), and colonies were screened for correct orientation of the gene. Selected colonies were grown in Blasticidin liquid media (InvivoGen, Toulouse, France) after which the isolated plasmid (Midi prep kit, Qiagen, Venlo, The Netherlands) was sequenced (Baseclear, Leiden, The Netherlands) to confirm non-mutated clones.

### Selection of Amino Acids on TLR2 for Mutant Development

As negatively charged pectin (DM7) could inhibit TLR2, we analyzed amino acids lying close to the ligand binding site on TLR2. We chose R315, R316, R321, and K347 amino acids (R-arginine and K-lysine) as putative pectin binding site on TLR2. R321 and R347 were selected as these positively charged amino acids were involved in binding between TLR2 and TLR1 interfaces (35). R315 and R316 were selected as these amino acids were structurally close to the ligand binding site of TLR2. The structural proximity of these amino acids on TLR2 structure was confirmed using PyMOL program (Schrödinger, New York, NY, USA).

### Development of TLR2 Mutant Cell Lines

The TLR2 gene sequence was changed at amino acid locations R315, R316, R321, and K347. The positively charged amino acids arginine (R) and lysine (K) were mutated at above locations to express uncharged amino acid glutamine (Q). The mutants were designed to have R315, R316, Q321, Q347 (TLR2-RRQQ) and Q315, Q316, Q321, and Q347 (TLR2-QQQQ). The TLR2-RRQQ and TLR2-QQQQ were synthesized by GeneArt AG service (Regensburg, Germany) with the hemagglutinin (HA) tag gene on the C-terminal and cloned in pcDNA™ 3.1 (+) Hygro plasmid (Life Technologies, Carlsbad, CA, USA). The plasmid was expanded in One Shot TOP10 Chemically Competent *E. coli* (Life Technologies, Carlsbad, CA, USA) and purified using Midi prep kit (Qiagen, Venlo, Netherlands). The plasmid was then linearized with Mfe I fast digest enzyme (Thermo Scientific, Waltham, MA, USA). The linearized plasmid was then transfected in HEK-Blue™ Null1 (InvivoGen, Toulouse, France) using Lipofectamine LTX^®^ (Life Technologies, Carlsbad, CA, USA).

### Plasmid Transfection for Stable Cell Line Development

HEK-Blue™ Null1 TLR2, HEK293T TLR2ectodomain-HA, HEK-Blue™ Null1 TLR2-RRQQ, and HEK-Blue™ Null1 TLR2-QQQQ cell lines were obtained by transfecting cells with NotI (Thermo Scientific, Waltham, MA, USA) linearized plasmids. Plasmids, antibiotics, and parental cell lines are listed in Table 2. The parental cell line was seeded at 500,000 cells/ml in 12-well culture plates and incubated overnight. The following day, transfection was performed using Lipofectamine LTX^®^ (Life Technologies, Carlsbad, CA, USA). Purified, 1 µg linear plasmid was diluted in low serum media Opti-MEM^®^ (Life Technologies, Carlsbad, CA, USA) and mixed with 3.5 µl of Lipofectamine LTX^®^ (Life Technologies, Carlsbad, CA, USA). This transfection mix was incubated for 30 min at room temperature and then added to the previously seeded cells in the culture media. Cells were incubated with transfection medium mix for 24 h, and transfected cells were selected using antibiotics. Single cell clones were selected for each newly developed cell line.

**Table 2:** Parental cell lines, expression plasmids and selection antibiotics used to develop toll-like receptor 2 (TLR2) expression cell lines.

Name of the cell line	Parental cell line	Plasmid for transfection	Selection antibiotics (InvivoGen)
HEK293T TLR2ectodomain-HA	HEK293T	TLR2ectodomain in pSELECT-CHA blasti	Blasticidin (50 µg/ml)
HEK-Blue™ Null1 TLR2	HEK-Blue™ Null1	pUNO3-hTLR2 (InvivoGen)	Zeocin (100 µg/ml) and hygromycin B (150 µg/ml)
HEK-Blue™ Null1 TLR2-RRQQ	HEK-Blue™ Null1	pcDNA™3.1 (+) Hygro plasmid-TLR2 RRQQ	Hygromycin B (200 µg/ml) and Zeocin (100 µg/ml)
HEK-Blue™ Null1 TLR2-QQQQ	HEK-Blue™ Null1	pcDNA™3.1 (+) Hygro plasmid-TLR2 QQQQ	Hygromycin B (200 µg/ml) and Zeocin (100 µg/ml)

### TLR Activation and Inhibition Assay

HEK-Blue™ cell lines were seeded at 500,000 cells/ml in 96-well plates at 100 μl/well. Cells were allowed to grow overnight. The following day, to study whether pectins can activate TLRs, cells were treated with different pectins at 2 mg/ml or TLR agonists as control (Table 1). Inhibition of TLR was studied by pretreating HEK-Blue™ cells with pectin (0.5, 1, or 2 mg/ml) for 1 h, followed by treatment with TLR agonists (Table 1). Activity of SEAP converts the pink QUANTI-Blue substrate to blue. Media supernatant was mixed with QUANTI-Blue in a ratio of 1:10 and NF-κB activation was quantified at 650 nm using a Versa Max ELISA plate reader (Molecular Devices, Sunnyvale, CA, USA). The assay was performed with five technical repeats. Each experiment was at least repeated three times.

### Dialysis Binding Assay of Pectin with TLR Agonist

Pectin was dissolved in 1 mM CaCl_2_ and 150 mM NaCl in 0.05 M Tris buffer pH 8.2 at 0.5, 1, and 2 mg/ml. Rhodamine-labeled Pam3CSK4 (InvivoGen, Toulouse, France) was added at 10 µg/ml and incubated overnight at 37°C. After incubation, 100 µl of this solution was applied to a 10 kDa molecular weight cut off microdialysis chamber (Thermo Scientific, Waltham, MA, USA). As pectin is larger than 10 kDa, it does not diffuse out of the microdialysis chamber, while the smaller 2.15 kDa Pam3CSK4 will diffuse out. In the case of an interaction between the TLR2 ligand Pam3CSK4 and pectin, the complex will stay inside the dialysis chamber and fluorescence will increase. The microdialysis chamber was washed with 1 mM CaCl_2_ and 150 mM NaCl in 0.05 M Tris buffer pH 8.2. Samples were dialyzed for 4 h, with buffer change every hour. After dialysis, samples were recovered from the chamber and fluorescence intensity was measured at 566 nm (excitation at 549 nm) using a Varioskan reader (Thermo Scientific, Waltham, MA, USA). Non-dialyzed and dialyzed Pam3CSK4 rhodamine in buffer was used as controls. Dialysis was performed three times for each test sample.

### Immunofluorescence

Human monocytic THP-1 cells (American Type Culture Collec-tion) were cultured in RPMI 1640 medium (Lonza, Belgium) with 10% fetal bovine serum (Sigma-Aldrich, MO, USA), 2 mM l-glutamine (Lonza, Belgium), 1 mM sodium pyruvate (Lonza, Belgium), 0.05 mM 2-mercaptoethanol (Scharlau, Spain), 60 µg/ml gentamicin sulfate (Lonza, Belgium), and 2.2 µg/ml amphotericin B solubilized (Sigma-Aldrich, MO, USA). THP-1 cell differentiation was induced by stimulation of THP-1 cells (1 × 10^6^ cells/ml) with 100 ng/ml Phorbol 12-myristate 13-acetate (PMA, Sigma-Aldrich) in an 8-wells chambered coverglass (Thermo Scientific, Nunc Lab-Tek, USA) for 48 h at 37°C and 5% CO_2_. The adherent cells were washed with PBS (Westburg, Netherlands) to remove PMA and they were then fixed for 15 min. with 4% paraformaldehyde (Merck, Germany). Subsequently, the cells were blocked with 10% goat serum (Sigma-Aldrich, MO, USA) for 1 h. The cells were incubated with the TLR2.1 FITC-conjugated antibody (1:200; ab13553, Abcam) for 1 h. Next, the cell was treated for 24 h with PBS, DM7 pectin, or DM75 pectin (1 mg/ml). After pectin treatment, the cells were incubated for 1 h with a rat anti-pectin primary antibody, specific for DM7 pectin (1:200; LM19, PlantProbes, University of Leeds, UK) or DM75 pectin (1:200; LM20, PlantProbes, University of Leeds, UK). Next, the cells were incubated for 30 min. with the secondary antibody for pectin goat-anti-rat Alexa Fluor 594-conjugated (1:500; Life Technologies, USA). DAPI (1.0 ug/ml; Roche, Switzerland) was incubated for 1 min on the cells and used as counterstain. In the end, the cells were covered with 250 µl PBS. The cells were washed thrice with 1× PBS between each incubation step, and all incubation steps were performed at 37°C and 5% CO_2_. Fluorescent confocal microscopy was measured using a Leica TCS SP8 AOBS confocal microscope (Leica Microsystem, Germany) equipped with an objective HC PL APO CS2 40×/0.30, immersion oil. All pictures were zoomed 6.00×. Data were processed and merged using ImageJ 1.47 Software (NIH, USA).

### Protein Immunoprecipitation

HEK293T TLR2ectodomain-HA, HEK-Blue™ Null1 TLR2-RRQQ, and HEK-Blue™ Null1 TLR2-QQQQ cells were lysed using RIPA lysis buffer (Merck Millipore, Billerica, MA, USA) with a protease inhibitor cocktail (Sigma, St. Louis, MO, USA) at 4°C for 10 min followed by two times sonication (Bandelin, Berlin, Germany) for 5 s at 50% power. Supernatant was collected after centrifugation at 14,000 *g* for 10 min. HA tagged proteins were immunoprecipitated using Pierce^®^ Anti-HA Agarose in a microcentrifuge tube. Protein was eluted using HA peptide (Thermo Scientific, Waltham, MA, USA) by incubating two times for 15 min with single bed volume of HA peptide at 30°C. Isolated protein was desalted and HA peptide was removed using Zeba Spin Desalting Columns and Devices, 40 K MWCO (Thermo Scientific, Waltham, MA, USA). Isolated desalted protein was quantified using the Thermo Scientific BCA protein assay kit (Thermo Scientific, Waltham, MA, USA).

### ELISA for Binding of TLR2 and Pectin

ELISA buffer consisted of 1 mM CaCl_2_ and 150 mM NaCl in 0.05 M Tris buffer at pH 8.2. The buffer was used for washing as well as diluent for antibodies and pectin. Blocking buffer was made by adding 3% milk powder (FrieslandCampina, Amersfoort, The Netherlands) to the ELISA buffer. For antibody solutions, 1:2 dilution of blocking buffer with ELISA buffer was used. ELISA plates (Corning, Tewksbury, MA, USA) were treated with 50 µl of 50 µg/ml of poly-l-lysine for 1 h at 37°C. Wells were washed once with 400 µl ELISA buffer. Pectins were dissolved in ELISA buffer at 1 mg/ml and 50 µl was applied to each well. The plates were incubated for 4 h at 37°C. Each well was then washed with 400 µl buffer and blocked overnight with 100 µl blocking buffer at 4°C. After blocking, the plate was washed once with ELISA buffer. Isolated TLR2 protein and HA peptide (Thermo Scientific, Waltham, MA, USA) were applied to pectin-coated wells at 0.33, 1, 3, and 9 µg/well; and incubated at 37°C for 3 h. HA peptide was used as a negative control. Pectin binding antibodies LM19 (DM7) and LM20 (DM22, DM45, DM60, and DM75) (Plantprobes, Leeds, UK) were used as positive control for pectin binding at 1:100 dilutions, to confirm even pectin immobilization. Afterward, wells were washed with 400 µl of ELISA buffer for five times and incubated with 50 µl primary HA tag antibody (Cell Signaling, Danvers, MA, USA) at 1:200 dilutions. Primary antibody was incubated for 2 h at 37°C. Afterward, plates were washed five times with ELISA buffer and subsequently, 50 µl of biotin tagged secondary antibody (Southern Biotech, Birmingham, AL, USA) was applied to each well at 1:500 dilutions. Biotin tagged antibody was incubated for 1 h at 37°C followed by five washings with 400 µl of ELISA buffer. Streptavidin-HRP (100 µl) was applied to each well at 1:1,000 dilutions. Anti-rat HRP antibodies (Cell Signaling, MA, USA) (1:500 for 1 h at 37°C) were used to detect pectin binding antibodies in control wells. In the final step, 100 µl TMB (Cell Signaling, MA, USA) was added as substrate for quantification and incubated at 37°C for 30 min. The reaction was stopped by addition of 100 µl of stop solution (Cell Signaling, MA, USA). Read out was done in a Versa Max plate reader (Molecular Devices, Sunnyvale, CA, USA) at 420 nm. The pectin loading control was confirmed to be at similar levels for all the pectin.

### Doxorubicin-Induced Ileitis in Mice

C57BL/6 female mice (7–10 weeks) were obtained from Janvier Laboratories, France. The experimental use of animals was approved by the Animal Ethical Committee of the University of Groningen. All the mice were acclimatized for 2.5 weeks before start of the experiment. Mice were fed *ad libitum* with RMH-B (AB Diets, Woerden, The Netherlands). The ingredients of the diet specified by supplier were as follows: wheat, meat meal (80% sterilized), yellow dent corn, whole oats, wheat middlings, alfalfa, soya oil, dried yeast, dicalcium phosphate, calcium carbonate, NaCl, dl-methionine, vitamins, and trace elements. As pectin is present mainly in fruits and vegetables, the basal of level of pectin in diet was minimal. Mice were supplied with drinking water from the tap and the water bottles were changed once a week.

Ileitis was induced by administration of doxorubicin (Sigma, St. Louis, MO, USA). Doxorubicin was dissolved in sterile 0.9% sodium chloride and stored in aliquots at 4°C. Pectin was dissolved in sterile water and administered by gavage to mice for 10 days, twice a day at a dose of 1.5 mg per gavage. On day 8, doxorubicin was injected intraperitoneally (IP) at 10 mg/kg. Mice were sacrificed on day 10 (48 h after doxorubicin). Animals receiving water by gavage served as controls. After the collection of tissue samples, mice were sacrificed by cervical dislocation.

Toll-like receptor 2 blocking antibody, clone T2.5 (InvivoGen, Toulouse, France) was administered IP at 10 mg/kg 1 h before doxorubicin treatment. The dose and concentration of TLR2 blocking antibody were used in accordance with Tye et al. (36). TLR2 blocking antibody was also administered in additional controls to study possible immune effects of TLR2 blocking antibodies in mice.

### Neutrophil Counts in Peritoneal Lavage

Peritoneal lavage was collected by injection and aspiration of 2 ml PBS. The total number of living cells in the peritoneal lavage, lysed with lyserglobin, was counted using a Z™ Series coulter counter^®^ (Beckman Coulter, Brea, CA, USA). The lavage was diluted to 500,000 cells/ml, and 100 µl of cell solution was applied for cytospin preparation. The cytospin slides were stained with Giemsa (Merck Millipore, Billerica, MA, USA) for 1 h at room temperature. The stained slides were scanned using a Hamamatsu slide scanner (Hamamatsu Photonics, Hamamatsu, Japan), and neutrophils were counted using morphological features in 250 cells (37). The total number of neutrophils was calculated using the total cell count of the peritoneum and the neutrophil counts from cytospin preparations.

### Histology

Ileal samples from mice were fixed in 10% formalin in PBS and embedded in paraffin. Paraffin blocks were cut in 4 µm sections. The tissue sections were analyzed for apoptosis in crypts using a TUNEL assay. The TUNEL assay was performed using the ApopTag^®^ Peroxidase *In Situ* Apoptosis Detection Kit (Merck Millipore, Billerica, MA, USA) according to the instructions of the manufacturer. Hematoxylin was used for counterstaining the slides. As peroxidase substrate, an incubation step of 15 min with 3-amino-9-ethylcarbazole (AEC) (5% AEC stock; 95% 0.05 M acetate buffer pH 4.9; 0.1% of 30% v/v H_2_O_2_) (Merck Millipore, Billerica, MA, USA) was used. The stained slides were scanned with a Hamamatsu slide scanner (Hamamatsu Photonics, Hamamatsu City, Japan), and analysis was performed using NDP.view2 Software. TUNEL positive cells were measured in 10 sequential crypts in the ileum per mouse.

### Cytokine Measurement Using Luminex Assays and ELISA

Peritoneal lavage from mouse was centrifuged to remove the immune cells and the supernatant was stored at −20°C. Blood was collected from mice through heart puncture and stored on ice in EDTA coated tubes (Greiner Bio-One, Kremsmünster, Austria). The tubes were centrifuged at 1,350 *g* for 10 min to separate plasma from the blood and stored at −20°C. Peritoneal lavage and plasma samples were analyzed in a luminex assay. TNF-α, MCP-1, IL-6, IP-10, and GRO-α were analyzed in the luminex assay (Affymetrix, Santa Clara, CA, USA).

Human umbilical cord dendritic cells (MatTek, Ashland, MA, USA) were seeded in a 96-well culture plates at 100,000 cells/ml in 100 µl dendritic maintenance medium provided by the supplier (MatTek, Ashland, MA, USA). After 24 h at 37°C, each well was treated with DM7 or DM75 pectin at 1, 10, and 100 µg/ml, dissolved in culture media. Similarly, TLR2 blocking antibody (InvivoGen, Toulouse, France) was applied as positive control at 10 µg/ml. Non-treated dendritic cells were used as negative control. After 1 h of pretreatment, 10 ng/ml of Pam3CSK4 was added. Dendritic cells treated with Pam3CSK4 or pectin only were used as control. After 24 h incubation, media supernatant was used to quantify IL-6 and IL-10 using the luminex assay (Affymetrix, Santa Clara, CA, USA).

RAW264.7 cells were seeded in 24-well plates at 10^6^ cells/well in RPMI medium with 10% FCS at a total volume of 500 µl. After 24 h at 37°C, each well was treated with DM7 or DM75 pectin at 1, 10, and 100 µg/ml dissolved in culture media. TLR2 blocking antibody (InvivoGen, Toulouse, France) was applied as positive control at 10 µg/ml. Non-treated RAW264.7 were used as negative control. After 1 h of pretreatment with pectin or TLR2 blocking antibody, 20 ng/ml of Pam3CSK4 was added. RAW264.7 treated with Pam3CSK4 or pectin only were used as control. After 24 h incubation, media supernatant was used to quantify IL-6 and IL-10 using ELISA (R&D Systems, MN, USA). ELISA was performed according to instructions from manufacturer.

The luminex assay was performed according to instructions from manufacturer. The antigen standards provided with the luminex kit (Affymetrix, Santa Clara, CA, USA) were dissolved and diluted fourfold to have seven serially diluted standards. DC-MM (MatTek, MA, USA) culture media was used as blank. The magnetic beads were added to a clear base, black 96-well plate at 50 µl/well and washed with a hand-held magnetic plate holder with 150 µl of the wash buffer provided in kit. The plasma samples were diluted with universal assay buffer in 1:2 dilution. The standards and samples in duplicates were applied to the magnetic beads, mixed on a plate shaker and incubated overnight at 4°C on a stable flat surface. The following day, magnetic beads were washed three times as mentioned earlier and incubated with 25 µl/well of detection antibody mix for 30 min on a plate shaker at room temperature. The plate was washed three times and incubated with 50 µl/well streptavidin-PE for 30 min at room temperature on a plate shaker. In the last step, the 96-well plate was washed three times and beads were dispersed in 120 µl of reading buffer per well and read in a Luminex-100 instrument with StarStation software.

### Organic Acids and SCFA Measurement

Wet digests from cecum (200 mg) and colon of mice was mixed with 1.5 ml of 0.3 mg/ml 2-ethylbutyric acid (internal standard) in 50 mM H_2_SO_4_. After centrifugation (10,000 *g*, 5 min, 20°C), the supernatant was transferred into a vial for injection using an HPLC method as described (38).

### Statistics

The results were analyzed using Graphpad Prism program (La Jolla, CA, USA). The parametric distribution of data was confirmed using Kolmogorov–Smirnov test. Values are expressed as mean ± SD except where nonparametric, in which case median ± range was used. Statistical comparisons were performed using two-way ANOVA for grouped analysis of parametrically distributed data. Where no parametric distribution could be demonstrated, we applied Mann–Whitney *U*-test or Kruskal–Wallis test. *p* < 0.05 was considered as statistically significant (*p < 0.05, **p < 0.01, and ***p < 0.001).

## Results

### Pectin Inhibits TLR2 Signaling in a Degree of Methyl Esterification and Concentration-Dependent Manner

**Figure 1:**
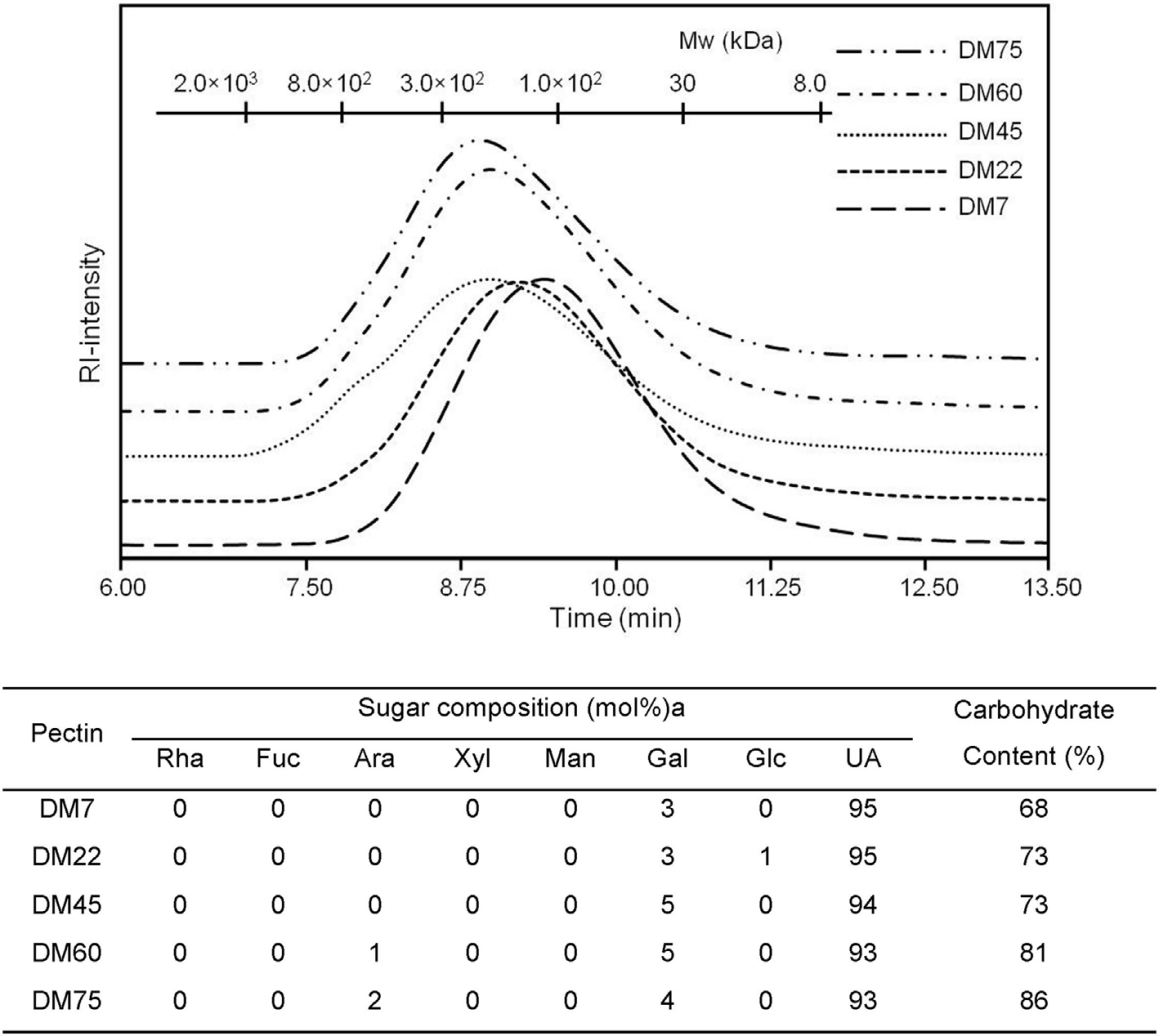
High pressure size exclusion chromatography elution patterns of pectin with monosaccharide composition (mol%) and carbohydrate content (w/w%).

**Figure 2:**
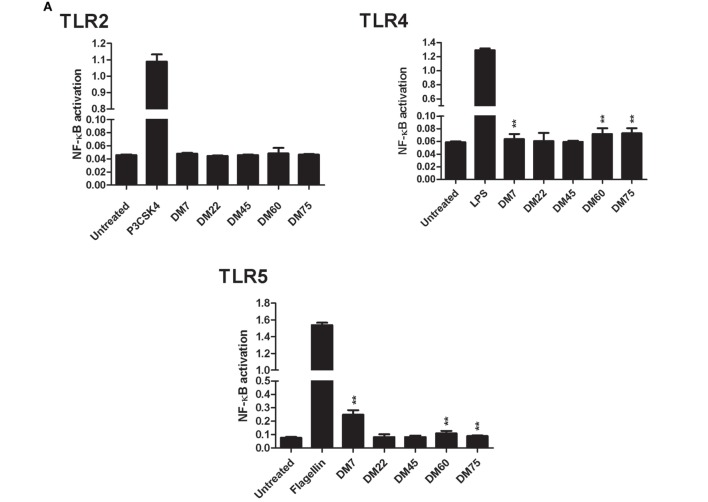

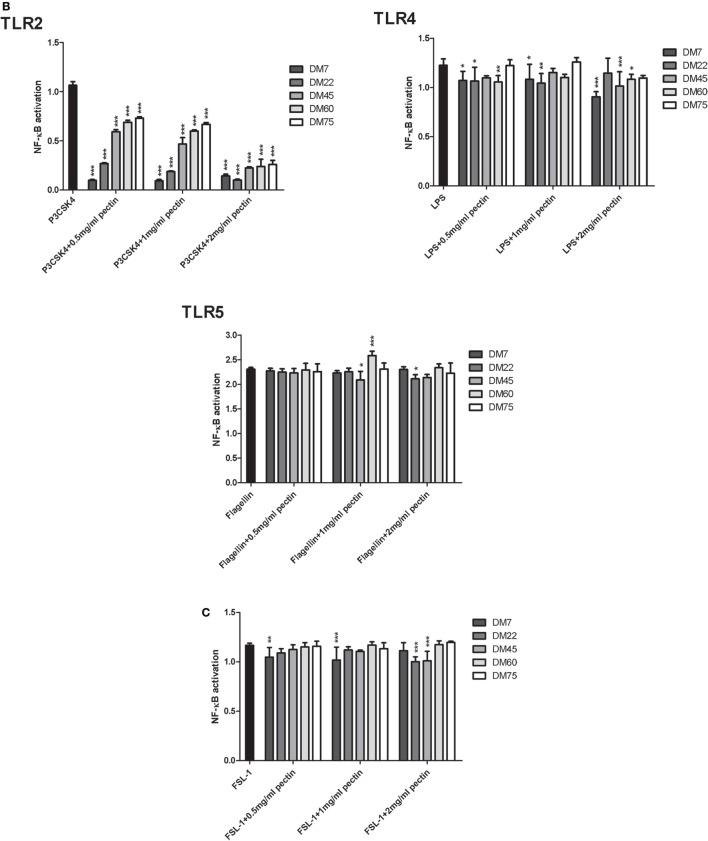
Pectin inhibits toll-like receptor 2 (TLR2)–TLR1, but not TLR2–TLR6, in a DM-dependent manner. **(A)** Toll-like receptor (TLR) activation represented as NF-κB activation of HEK-Blue™ TLR stimulated with pectin at 2 mg/ml concentration and with P3CSK4, LPS, and Flagellin as positive control for TLR2, TLR4, and TLR5 activation, respectively (*n* = 5). Data are presented as median ± range. The statistical differences between pectin samples were quantified using Kruskal–Wallis test. **(B)** TLR inhibition in HEK reporter cell lines for TLR2, TLR4, and TLR5, which were pretreated with pectin followed by activation by P3CSK4, LPS, and Flagellin, respectively (*n* = 5). **(C)** TLR2–TLR6 inhibition by pectin in HEK TLR2 reporter cells, which were pretreated with pectin and followed by stimulation with FSL-1 (*n* = 5). Data are presented as the mean ± SD, and statistical significance was calculated using two-way ANOVA analysis (*p < 0.5, **p < 0.01, and ***p < 0.001).

To study the effects of chemical composition of pectin on the immune properties, we chemically characterized citrus pectin with different degrees (percent) of methyl esterification (DM7, DM22, DM45, DM60, and DM75) and studied activation and inhibition of TLR signaling. The pectins had average molecular weights ranging from 50 to 300 kDa and were predominantly composed of d-galacturonic acid (Figure 1). To determine TLR activation by pectins, the pectins were incubated with HEK293 NF-κB reporter cell lines expressing extracellular TLRs (TLR2, TLR4, and TLR5). TLR activation with specific ligands in reporter cell lines results in NF-κB activation, which can be quantified through expression of SEAP gene transcribed through an NF-κB controlled promoter. As shown in Figure 2A pectins were unable to activate TLRs. It was never more than 10% of the positive controls (Figure 2A).

Pectins had a pronounced inhibitory effect on specific TLR receptors (Figure 2B) Inhibitory effects of pectin were studied by adding a TLR agonist to TLR reporter cell lines pretreated with pectin. As shown in Figure 2B; Figure S2 in Supplementary Material, pectin strongly inhibited TLR2 activation and minimally inhibited TLR4 and TLR5. There was a clear DM-dependent effect, as inhibition of TLR2 by lower DM pectins was more pronounced than with pectins of higher DM. Pectin DM7 suppressed TLR2 activation by 91.1 ± 0.8% (*p* < 0.001) at the lowest concentration tested (0.5 mg/ml) (Figure 2B). Increasing the concentration enhanced the efficacy of higher DM pectins to inhibit TLR2 (Figure 2B).

### Pectin Inhibits TLR2–TLR1 Activation but Not TLR2–TLR6

Toll-like receptor 2 is activated by forming heterodimers with either TLR1 or TLR6. TLR2 activation with either TLR1 or TLR6 stimulates different pathways (30). As such, the TLR2 reporter cell line also expresses TLR1 and TLR6 (39). TLR2–TLR1 is specifically activated by a tri-acylated lipopeptide, P3CSK4 agonist. As shown in Figure 2B, the TLR2–TLR1 dimer was inhibited by pectin in a DM- and concentration-dependent manner. The other heterodimer, TLR2–TLR6, is activated by diacylated lipopeptides, such as FSL-1 (40). To determine if pectin also inhibits TLR2–TLR6 activation, different DM pectins were evaluated with FSL-1 as an agonist. As shown in Figure 2C, pectin did not substantially inhibit TLR2–TLR6. Thus, pectin only inhibits TLR2–TLR1 but does not inhibit TLR2–TLR6 activation.

### Pectin Inhibits TLR2 by Direct Binding to TLR2 Ectodomain by Electrostatic Interactions

We performed four experiments to confirm the interaction between TLR2 and pectin. In the first experiment, we excluded the possibility that TLR2 inhibition was due to binding of pectin to the ligand P3CSK4. In the second and third experiment, we studied direct binding between pectin and TLR2 by immunofluorescence and ELISA. In the fourth, we identified the sites on TLR2 involved in binding to pectin by generating TLR2 mutants at predicted binding sites.

**Figure 3:**
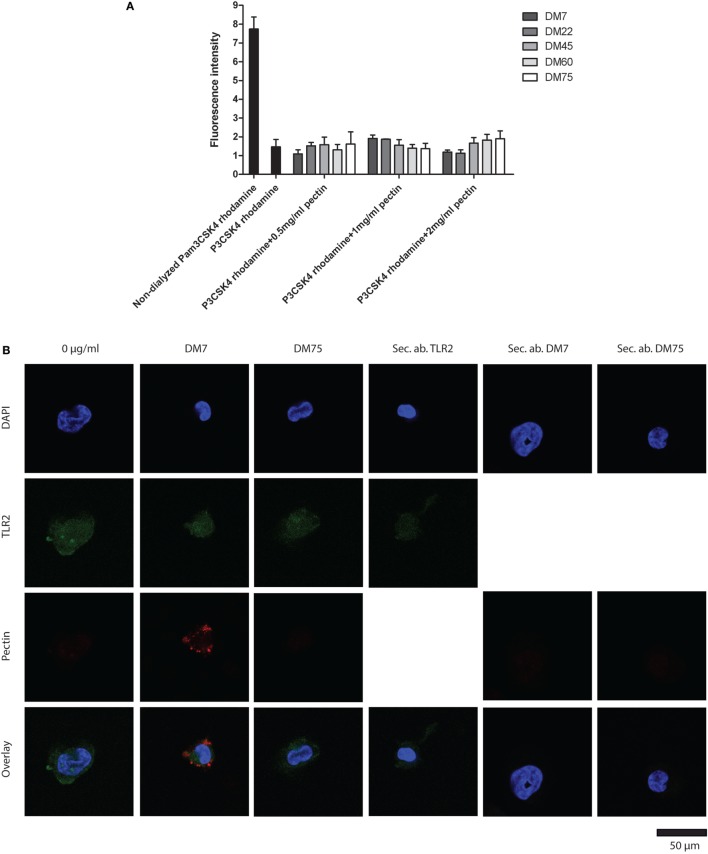

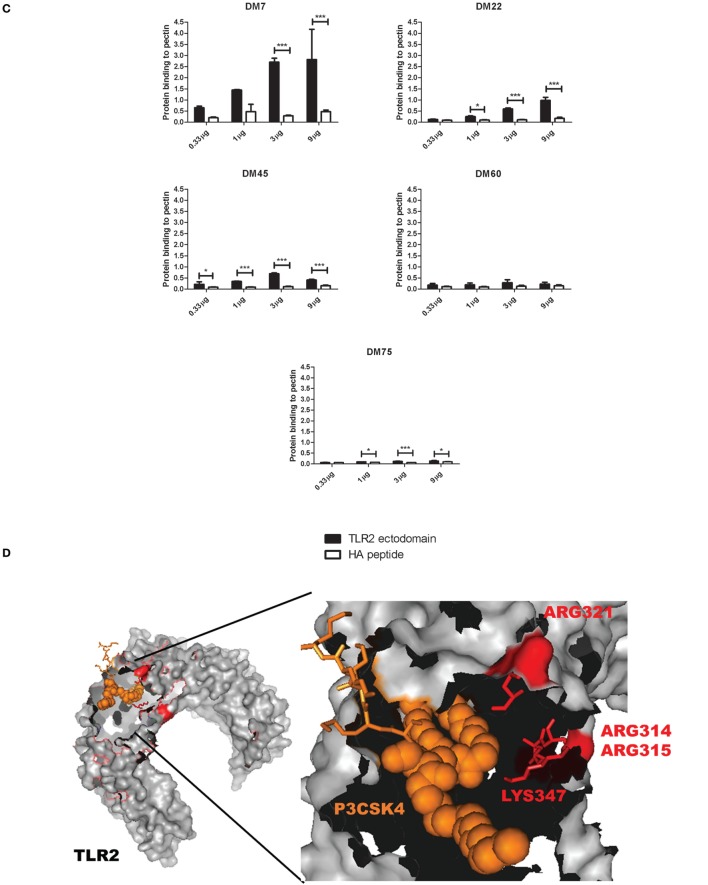

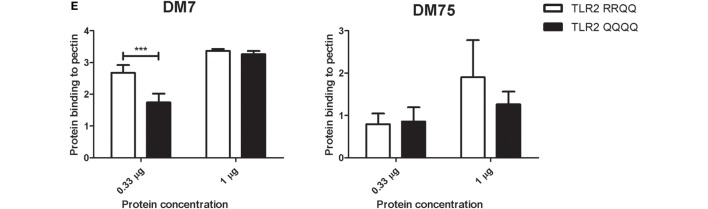
Low-DM pectin inhibits toll-like receptor 2 (TLR2)–TLR1 by directly binding to the TLR2 ectodomain through electrostatic interactions **(A)** P3CSK4 rhodamine interactions with pectin presented as fluorescence retention in the dialysis membrane compared with non-dialyzed samples (*n* = 3). **(B)** Immuno-fluorescence co-staining of TLR2 (green) with DM7 or DM75 pectin (red). Sec. ab are the secondary antibody controls for TLR2, DM7, and DM75 antibodies (*n* = 3). **(C)** TLR2 ectodomain binding to pectins with various DM. Isolated hemagglutinin peptide was used as a negative control (*n* = 3). **(D)** TLR2 crystal structure with P3CSK4 and R315, R316, R321, and K347. **(E)** TLR2 mutants, TLR2-RRQQ and TLR2-QQQQ binding to DM7 and DM75 pectin (*n* = 3). Data are presented as the mean ± SD, and statistical significance was calculated using two-way ANOVA analysis (*p < 0.05 and ***p < 0.001).

In the first experiment, pectin was co-incubated with fluorescently labeled P3CSK4 and dialyzed using a 10 kDa selective dialysis membrane permissible to P3CSK4 but not to pectin. As shown in Figure 3A, pectin did not bind P3CSK4, as fluorescence decreased profoundly upon dialysis. Also, there was no difference between fluorescence levels of dialyzed P3CSK4 with and without pectin. Thus, pectin does not bind to the TLR2–TLR1 agonist P3CSK4.

To determine direct binding between pectin and TLR2, we first studied the combined presence of pectin and TLR2 on the cell membrane of TLR2-expressing THP-1 macrophages by immunostaining. As shown in Figure 3B, we observed co-staining of TLR2 (green) and pectin (red). The differentiated THP-1 cells were treated with DM7 and DM75 pectin. We observed a bright staining of DM7 pectin on the cell surface as well as of TLR2. With DM75 pectin we found no staining on the cell surface illustrating its inability to bind to TLR2. To further confirm, we developed a specific ELISA assay to demonstrate binding of pectin to TLR2. To this end, we made cell lines expressing the TLR2 ectodomain consisting of the ligand binding site. It was C-terminally fused to an HA tag. The TLR2 ectodomain protein was extracted, purified and applied to a direct ELISA containing immobilized pectin (Figure S1 in Supplementary Material). As shown in Figure 3C, TLR2 binding to pectin was DM dependent, wherein lower DM pectins showed stronger binding to TLR2 while minimal binding was observed for high-DM75 pectin. Thus, we confirm that inhibition of TLR2 was by binding of pectin to TLR2 and that lower DM pectins can bind more readily to TLR2 than high-DM pectins.

To determine how and where low-DM pectins can bind to TLR2 we searched for potential binding sites on TLR2. As low-DM pectins are hydrophilic and more negatively charged than higher DM pectins we hypothesized that the positively charged amino acids at positions R315, R316, R321, and K347 surrounding the ligand binding site are responsible for interaction with low-DM pectin (Figure 3D). To test this, we developed TLR2 mutants wherein we substituted amino acids R315, R316, R321, and K347 to the uncharged amino acid glutamine (Q). R321 and K347 have been shown to be involved in TLR2 and TLR1 interface binding (35). R315 and R361 were mutated as these positively charged amino acids lie structurally in proximity of the ligand binding site of TLR2 (35). We developed two mutants wherein only R321 and K347 were changed to Q (RRQQ) or all the amino acids were changed to Q (QQQQ) to allow comparison of binding kinetics. As shown in Figure 3E, the DM7 pectin showed lower binding with TLR2-QQQQ than TLR2-RRQQ (*p* < 0.01). Whereas we did not observe any difference in binding of DM75 pectin with both of the TLR2 mutants. This confirms that the pectin interacts with TLR2 through electrostatic forces at the R315, R316, R321, and K347 sites. The higher level of binding observed to the TLR2 mutants compared with the TLR2 ectodomain might be due to the use of full-length TLR2 constructs in TLR2 mutants than in the TLR2 ectodomain.

### Pectin Inhibits TLR2–TLR1 in Human Dendritic Cells and in Mouse Macrophage Cell Line RAW264.7

**Figure 4:**
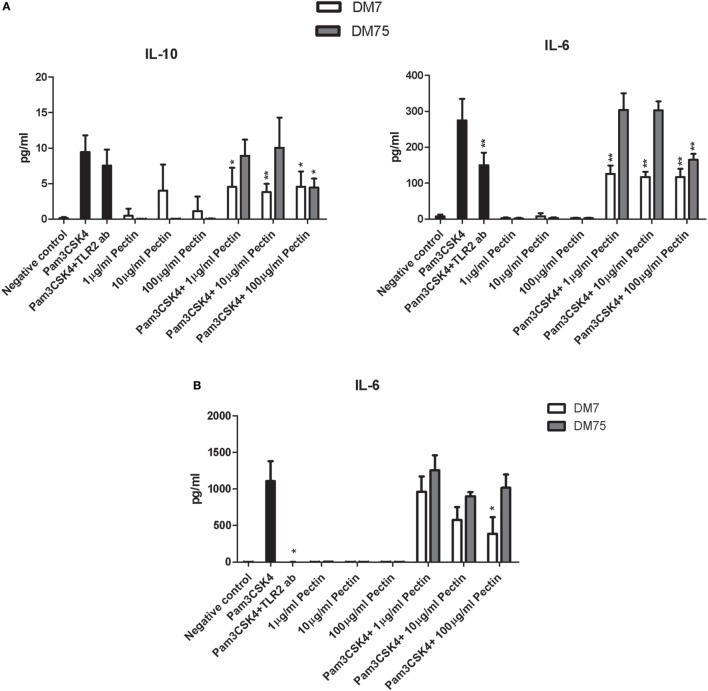
Pectin inhibits toll-like receptor 2 (TLR2)–TLR1 activation in human dendritic cells and mouse macrophage RAW264.7 cells. **(A)** IL-10 and IL-6 levels in human dendritic cells after P3CSK4 activation with or without pectin DM7 or DM75 pretreatment (*n* = 5). **(B)** IL-6 levels in RAW264.7 cells after P3CSK4 activation with or without pectin DM7 or DM75 pretreatment (*n* = 3). Data are presented as the mean ± SD, and statistical significance was calculated using the Mann–Whitney *U*-test (*p < 0.05 and **p < 0.01).

The effect of DM of pectin on TLR2–TLR1-dependent inhibition of immune responses was also studied in human dendritic cells and in the mouse macrophage cell line RAW264.7. Dendritic cells and RAW264.7 cells were stimulated with P3CSK4 in the presence of the TLR2 inhibiting pectin DM7 and a weak TLR2 inhibiting pectin DM75. Production of the P3CSK4-dependent cytokines IL-6 (41) and IL-10 (42) were used to assess the effects. Concentration- and DM-dependent effects were observed for TLR2 inhibition by pectin (Figure 4). In dendritic cells, at high concentrations (100 µg/ml), both DM7 and DM75 pectin reduced IL-6 and IL-10 production. However, at lower concentrations (10 and 1 µg/ml) only DM7 pectin reduced IL-6 and IL-10 production (*p* < 0.001). Remarkably, the inhibition potency of DM7 pectin was similar, if not identical, to that of a TLR2 blocking antibody (Figure 4A). In RAW264.7 cells, DM7 reduced P3CSK4 induced IL-6 production at 100 µg/ml, while DM75 was ineffective in reducing IL-6 production (Figure 4B). IL-10 production could not be induced in RAW264.7 cells using P3CSK4 (data not shown). Thus pectin DM7 can reduce P3CSK4 induced cytokine production in both human dendritic cells and in mouse macrophages.

### Pectin Is Protective against TLR2-Dependent Ileitis in Mice

**Figure 5:**
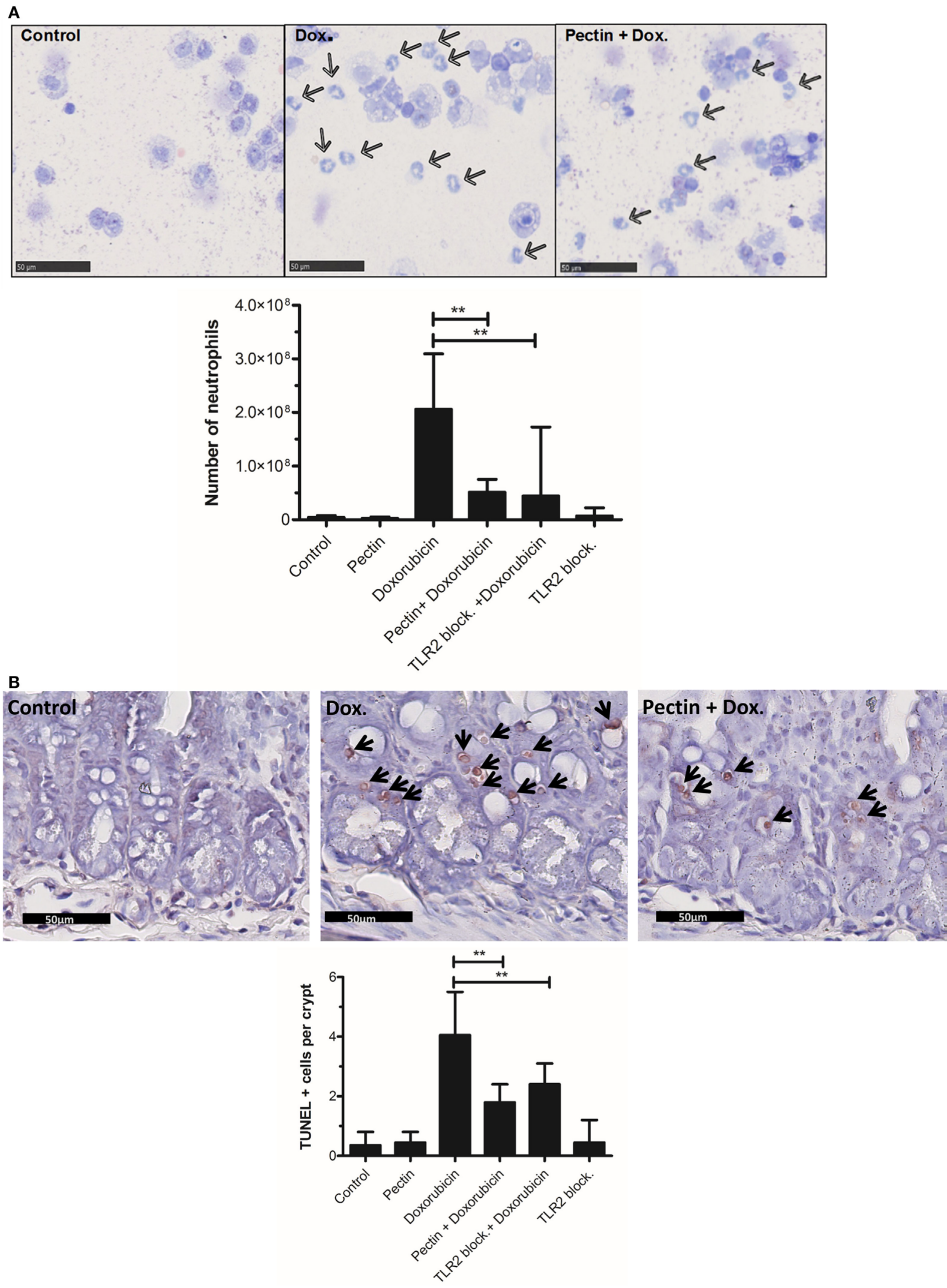

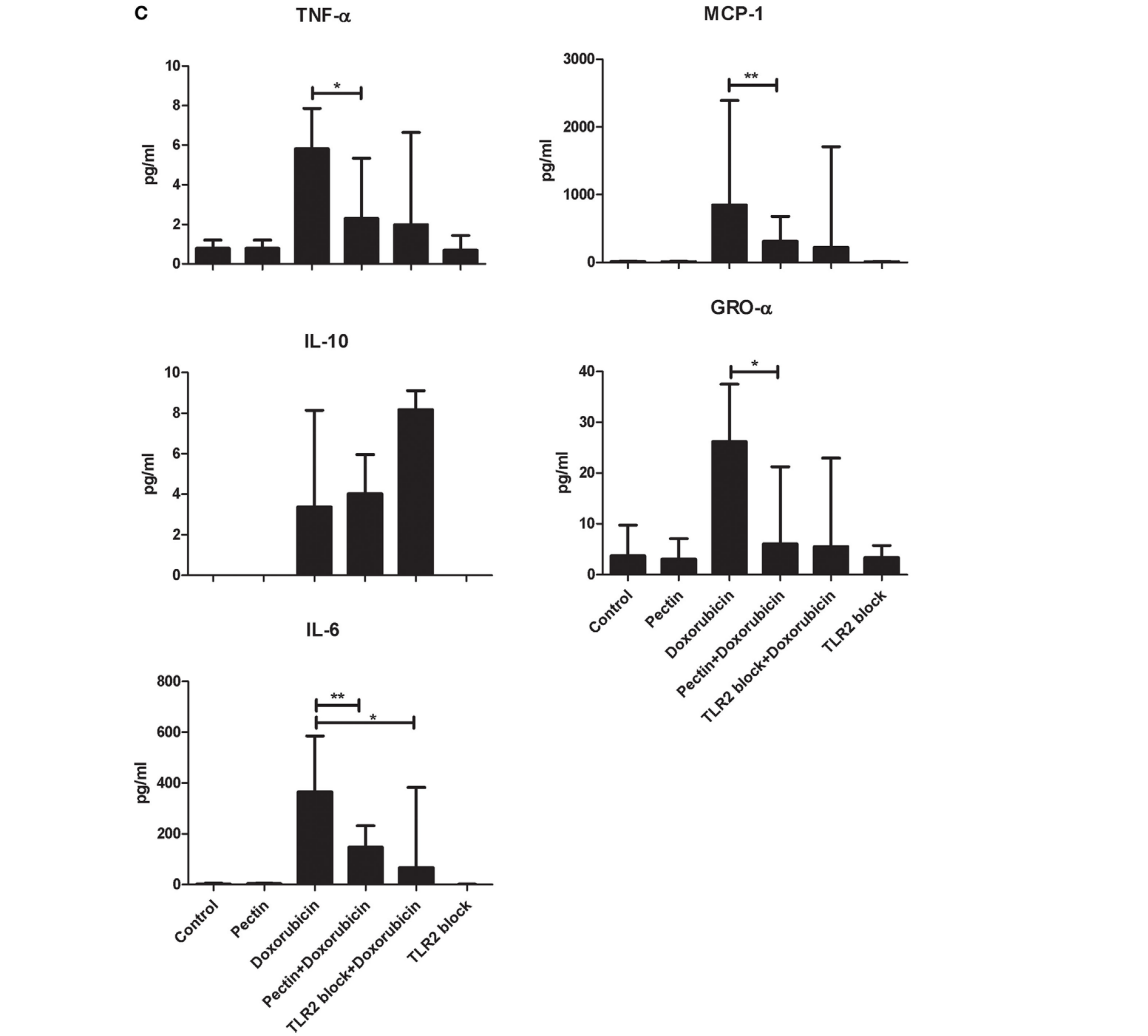
Low-DM pectin is protective against doxorubicin-induced ileitis through toll-like receptor 2 (TLR2)–TLR1 inhibition. **(A)** Neutrophils (indicated by arrows) in peritoneal lavage stained with Giemsa. **(B)** Ileal apoptotic cells (indicated by arrows) determined using the TUNEL assay. Stained cells were counted in 10 sequential crypts per mouse. **(C)** Cytokine and chemokine levels in peritoneal fluid (*n* = 6). Data are presented as the median ± range, and statistical differences were calculated using the Mann–Whitney *U*-test (*p < 0.05 and **p < 0.01).

To confirm efficacy of TLR2 inhibition by low-DM pectin *in vivo*, DM7 pectin was tested for suppression of TLR2-dependent ileitis induced by doxorubicin in a mouse model. Doxorubicin induces massive cell death in intestinal crypts and immune cells (37), which leads to the release of damage-associated molecular pattern molecules (DAMPs) that induce a strong TLR2-dependent inflammatory response which is subdued in TLR2 knockout mice compared with the wild-type mice (37). Pectin DM7 was administered for 1 week at 3 mg/day followed by doxorubicin treatment for 48 h. Pectin administration was continued until the animals were sacrificed. Initial symptoms of doxorubicin-induced ileitis included neutrophil influx in the peritoneum (43) and cell death in crypts of the small intestine (37), as shown in doxorubicin-treated controls (Figures 5A,B). Neutrophil influx and cell death in the crypts were statistically significantly lower in mice with doxorubicin-induced ileitis treated with pectin DM7 (*p* < 0.01) (Figures 5A,B) than in mice treated with doxorubicin only. Strong inhibitory effect of pectin DM7 on ileitis are illustrated by the reduction in neutrophil influx and cell death in the crypts that was similar to the reduction in mice treated with TLR2 blocking antibodies (*p* < 0.01) (Figures 5A,B).

Toll-like receptor 2–TLR1 activation has been reported to lead to proinflammatory responses (41, 44). The cytokine profile in peritoneal fluid of mice demonstrates that pectin indeed specifically inhibited the TLR2–TLR1-induced proinflammatory response. DM7 pectin treatment reduced production of the proinflammatory cytokines TNF-α (*p* < 0.05), MCP-1 (*p* < 0.01), GRO-α (*p* < 0.05), and IL-6 (*p* < 0.01) (Figure 5C), with a similar efficacy to that of TLR2 blocking antibodies in doxorubicin-treated mice. The immune regulatory cytokine IL-10 remained unchanged after pectin treatment in doxorubicin-treated mice (Figure 5C) suggesting that the regulatory pathway remained unaffected.

### Anti-inflammatory Effects of Pectin Are Not through Stimulated SCFA Production

**Figure 6:**
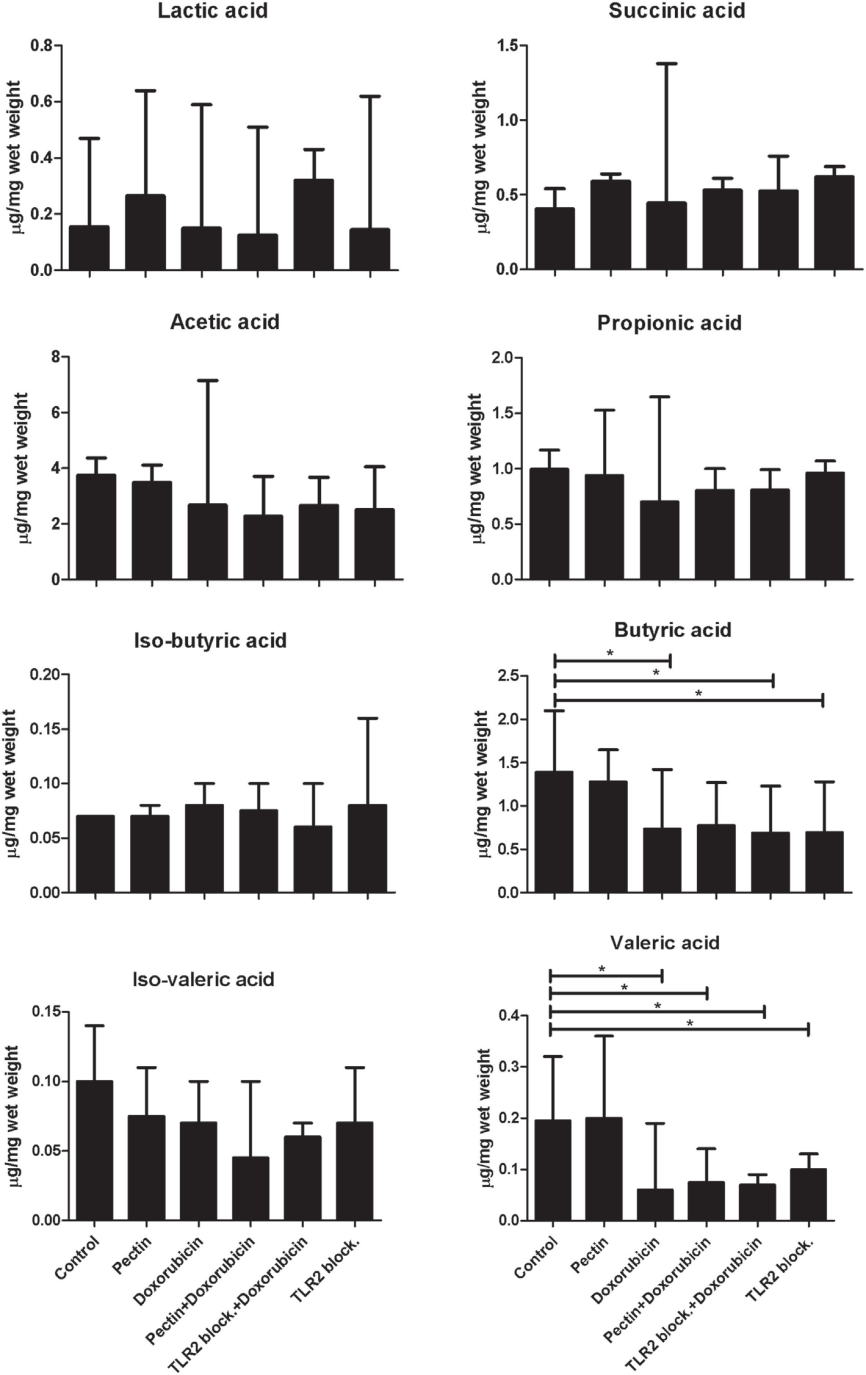
The anti-inflammatory effect of low-DM pectin is due to toll-like receptor 2 (TLR2)–TLR1 inhibition versus short-chain fatty acids (SCFAs). SCFA concentrations in cecum samples from mice after doxorubicin treatment with pectin (*n* = 6). Data are presented as the median ± range, and statistical differences were calculated using the Mann–Whitney *U*-test (*p < 0.05).

It might be suggested that attenuation of immune responses in mice may not be entirely caused by direct interaction with TLR2, but might be supported by anti-inflammatory SCFA production by microbiota that possibly metabolize pectin. To exclude this, we quantified the concentration of organic acids (lactic acid and succinic acid) and SCFAs (acetic acid, propionic acid, iso-butyric acid, butyric acid, iso-valeric acid, and valeric acid) in cecum samples from mice (Figure 6) after doxorubicin and/or pectin treatments. SCFAs were not enhanced and therefore cannot be responsible for attenuation of immune responses. Butyric and valeric acid were significantly reduced by doxorubicin treatment (*p* < 0.05), which was not prevented by pectin treatment (*p* < 0.05) (Figure 6). Administration of a TLR2 blocking antibody alone also reduced butyric and valeric acid levels (*p* < 0.05) (Figure 6), suggesting that these changes might be due to TLR2 blocking in the colon.

## Discussion

Despite intensive research and recognition of the importance of dietary fibers for human health (8), little is known about the mechanism or the structures on fibers responsible for immunomodulatory effects. Much attention has been focused on effects of microbial degradation products of dietary fibers such as immune regulatory SCFA (45, 46) but as shown here this cannot explain all the immune effects of widely consumed dietary fibers such as pectins which does not enhance SCFA but still exhibits a pronounced anti-inflammatory effect. Direct binding of dietary fibers and activation of PRRs has been suggested before as possible mechanism for immunomodulation (47), but inhibition of proinflammatory PRRs has to the best of our knowledge not been studied or suggested before.

Here, we show that the dietary fiber pectin can inhibit TLR2, attenuate immune responses *in vitro* and *in vivo*. This effect is dependent on DM of pectin. Also, we identified by applying targeted designed mutants of TLR2 the binding sites in TLR2 for low-DM pectin. In addition, we demonstrate the potential clinical applicability of the obtained insights by testing low-DM pectins in a well-established TLR2-dependent ileitis model (37) in which it blocked ileitis as efficacious as TLR2 antibodies. Our work provides novel insights into mechanisms of health promoting effects of dietary fibers and demonstrates how structure–function studies can assist in design of effective dietary fiber formulations.

Pectin as a dietary fiber has been demonstrated to have different immune effects depending on its chemical composition (18, 19, 48). One of the mechanisms for immunomodulation by pectin was suggested to be by inhibition of TLR4 (18, 19). However, the inhibition was by sequestering of LPS by pectin (18), and a true blocking of TLR4 receptor by pectin was not confirmed (18, 19). Recently, even stimulation of TLR4 by pectins has been reported (49). In our reporter cell line assays, we did not observe a substantial activation or inhibition of TLR4 which might be due to different size (19), composition or origin of pectin fibers used in our study compared with previous studies (18, 19, 48, 49). The lemon pectins used in our study are virtually of the same size, same origin, and only differ in the level of methyl esterifications, allowing sound comparison and demonstration of a clear structure–function relationship for immunomodulatory effects of pectin.

The TLR2 inhibition was both dependent on concentration and DM value of pectin. The low-DM pectin showed the highest efficiency in TLR2 inhibition at low concentrations. Whereas at high concentrations, both high- and low-DM pectin could inhibit TLR2 with similar efficiencies. The concentration-dependent effect can be explained by charge densities on pectins at different concentrations. Low-DM pectins have higher levels of unesterified regions and are more charged compared with high-DM pectins (50). At higher concentrations, high-DM pectins have more effective concentrations of unesterified galacturonic acid patches than at low concentrations. Thus, due to this higher effective concentration of unesterified regions on pectin, high-DM pectin inhibits TLR2 more efficiently at higher concentrations. Similar phenomena have also been observed in enhanced gel formation by high-DM pectin at increased concentrations due to more effective concentration of free carboxyl groups of galacturonic acids in pectin (50, 51).

The TLR2 inhibition by pectin was specific for TLR2–TLR1 and did not occur for TLR2–TLR6, although pectin is able to bind to TLR2 ectodomain. This difference may be due to the different kinetics of interaction forces between TLR2–TLR1 and TLR2–TLR6 interfaces. TLR2–TLR1 binding is governed both by ionic and hydrophobic forces (35), whereas binding at the TLR2–TLR6 interface is mainly through hydrophobic forces (52). Pectins, and especially low-DM pectins being highly charged molecules that are hydrophilic in nature (53), might therefore interfere with ionic TLR2–TLR1 interactions without hampering TLR2–TLR6 hydrophobic interactions. This argument is supported by our observation of DM-dependent binding of pectin to the TLR2 ectodomain (Figure 3C). The TLR2 ectodomain demonstrated a high binding of low-DM pectins but a minimal binding of high-DM pectins. This charge dependent binding of low-DM pectin with TLR2 was also observed with TLR2 mutant constructs. However, when comparing binding of pectin to the TLR2 ectodomain with that to the TLR2 mutants we observed a higher binding in TLR2 mutants which might be due to the full-length TLR2 construct used in mutants. The full-length TLR2 mutants contain in addition to the ectodomain also the intermembrane domain and the intracellular domain of TLR2 (35, 52). Presence of these extra domains in TLR2 mutants might enhance the capacity to bind to pectin. While comparing TLR2 mutants, we observed differences in mutants with different levels of charges. We showed that the positively charged amino acids surrounding the TLR2 ligand binding site are important for binding with low-DM pectin and not for high-DM pectin binding. This suggests that the binding between TLR2 and pectin is mainly due to electrostatic forces as TLR2 mutants having less positive charge, i.e., TLR2-QQQQ bound at lower affinity with negatively charged DM7 pectin while having similar binding affinity with DM75 pectin having lower charge densities.

The TLR2 inhibiting properties of low-DM pectin were further confirmed in doxorubicin-induced ileitis in mice. Doxorubicin-induced ileitis is reported to be prevented by blocking of TLR2 (37). Low-DM pectin administration in doxorubicin-induced inflammation in mice reduced the symptoms. Strikingly, the anti-inflammatory effects of pectin were as effective as TLR2 blocking antibody administration. The proinflammatory cytokine production in peritoneal fluid was reduced after pectin adminis-tration in mice treated with doxorubicin, while the regulatory IL-10 cytokine remained unchanged. Pectin administration did not enhance SCFA levels, again confirming that the effects were TLR2 inhibition dependent. TLR2 blocking antibody treatment alone showed reduction in butyric and valeric acid. The effects might be due to side effects of antibody treatment in cecum as this was not observed with pectin treatment. These observations substantiate the importance of dietary fibers like pectin as potential therapeutic anti-inflammatory molecules which might be useful in treating immunological disorders of intestinal origin. TLR2 signaling is responsible for inflammation in doxorubicin-induced ileitis. However, in other contexts, TLR2 signaling may be beneficial as TLR2 signaling has also been shown to induce tolerogenic immune responses through induction of regulatory T cells (54). For example TLR2 ligands, have been shown to be protective effects against autoimmune diseases such as multiple sclerosis (55). Thus, both inhibiting and activating dietary fibers may be instrumental in managing specific diseases and it is of utmost importance to determine mechanisms of anti-inflammatory effects of dietary fibers in specific contexts to benefit from dietary fibers.

**Figure 7:**
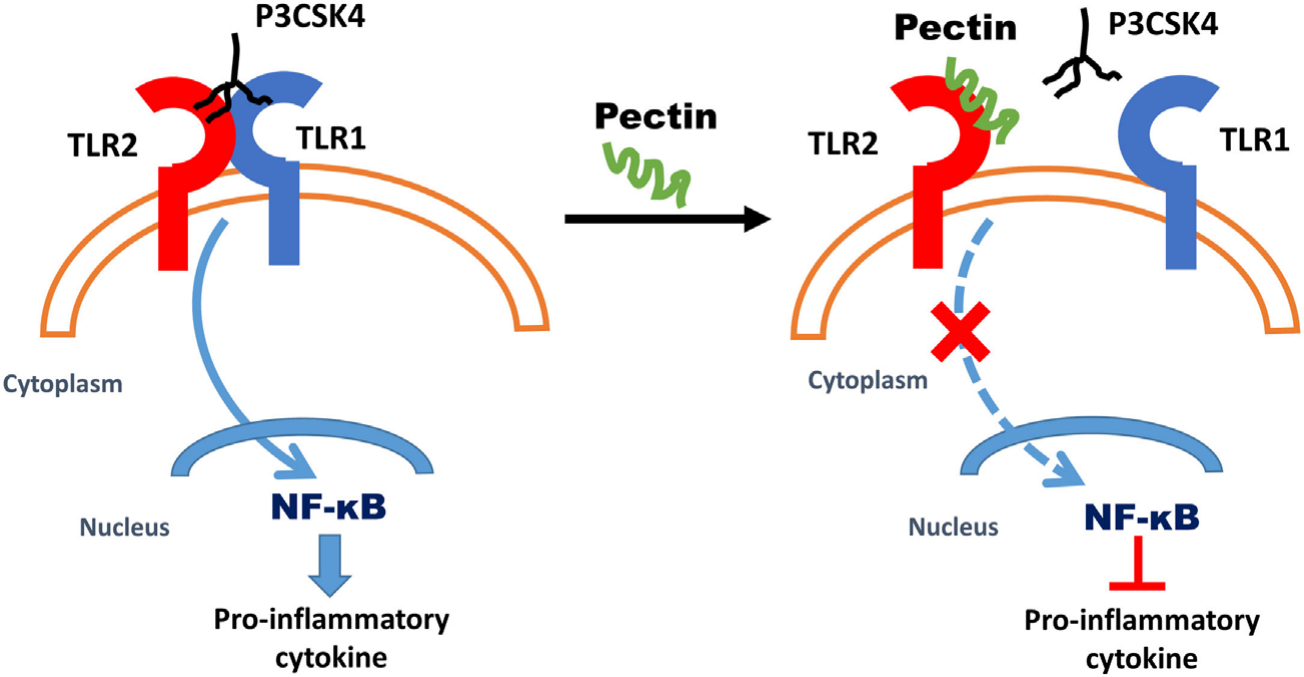
Proposed mechanism of anti-inflammatory effects due to low-DM pectin. Dietary fiber low-DM pectin binds to toll-like receptor 2 (TLR2), which results in inhibitions of TLR2–TLR1 heterodimer activation and thus reduced NF-κB activation.

In this study, we demonstrate that dietary pectins with a low DM can effectively bind to TLR2 at low concentrations and prevent proinflammatory responses, even in the presence of a TLR2–TLR1 stimulus. As pectin is specific for inhibition of TLR2–TLR1, the regulatory TLR2–TLR6 (44) heterodimer signaling remained intact, and thus tolerogenic immune responses remained unaffected. In Figure 7, we propose a mechanism by which pectins suppress TLR2–TLR1 by direct blocking of TLR2 receptor. TLR2–TLR1 inhibition by pectin was also effective in preventing an intestinal disorder in which intestinal inflammation is exacerbated by TLR2 activation. Inflamed intestinal lesions through release of DAMPs such as high-mobility group box-1, leading to proinflammatory cytokine release (56) and intestinal damage through TLR2 (57) was prevented by oral pectin administration. The anti-inflammatory effects of pectin *via* TLR2–TLR1 inhibition can be an effective strategy in rationally designing dietary fiber formulations to prevent or cure inflammation and may even provide an explanation as to why increased dietary fiber intake is associated with a lower frequency of immunological disorders (8, 48).

## Ethics Statement

The experimental use of animals was approved by the Animal Ethical Committee of the University of Groningen, DEC6669.

## Author Contributions

NS and PV conceptualized all the experiments and prepared the manuscript. The *in vitro* work was performed by NS, and immunofluorescence was performed by MB. The *in vivo* work was performed by NS and BT. LT and HS chemically characterized pectin, quantified SCFAs, and contributed to manuscript preparation. JS, EB, GB, MvdB, AS, and MF were involved in practical discussions and manuscript review.

## Conflict of Interest Statement

EB was employed by company Agrifirm, GB was employed by company NuScience, and MvdB was employed by company DSM. The remaining co-authors declare that the research was conducted in the absence of any commercial or financial relationships that could be construed as a potential conflict of interest.

## Supplementary Material

Figure S1 Direct ELISA to test binding of Toll-like Receptor 2 to pectin.

Figure S2 Pectin inhibits Heat killed Listeria monocytogenes (HKLM) stimulated Toll-like Receptor (TLR) 2. TLR inhibition in HEK reporter cell lines for TLR2 which were pretreated with pectin followed by activation by HKLM (n=5). Data are presented as the mean ± SD, and statistical significance was calculated using two-way ANOVA analysis (*p < 0.001).
