# Supplementary figures and images for: Dietary Fiber Pectin Directly Blocks Toll-Like Receptor 2–1 and Prevents Doxorubicin-Induced Ileitis
Source: Front Immunol. 2018 Mar 1;9:383. doi: 10.3389/fimmu.2018.00383 (PMC5839092; doi:10.3389/fimmu.2018.00383)

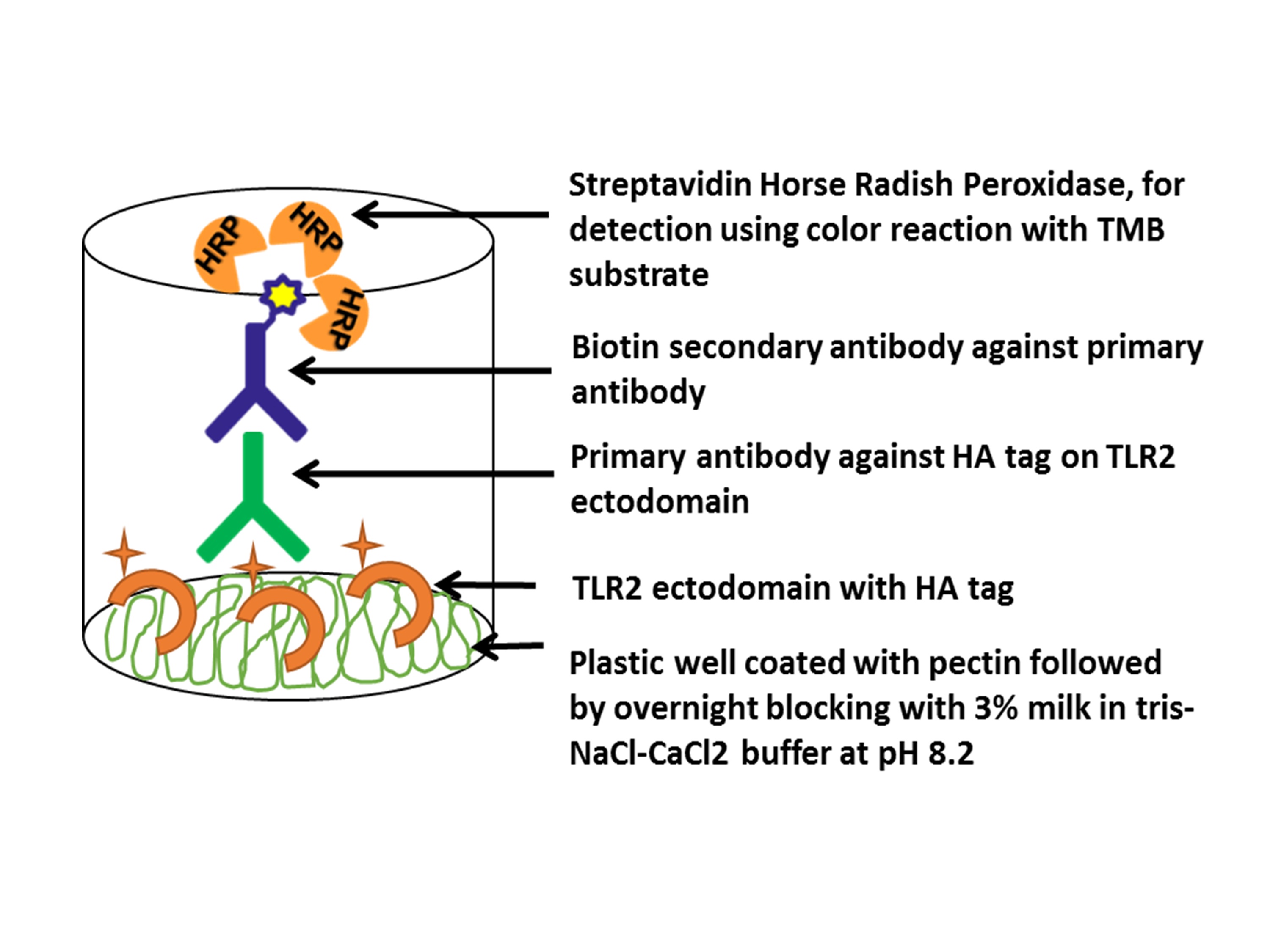

Supplement: Figure S1 — Direct ELISA to test binding of Toll-like Receptor 2 to pectin. [file image_1.tif]

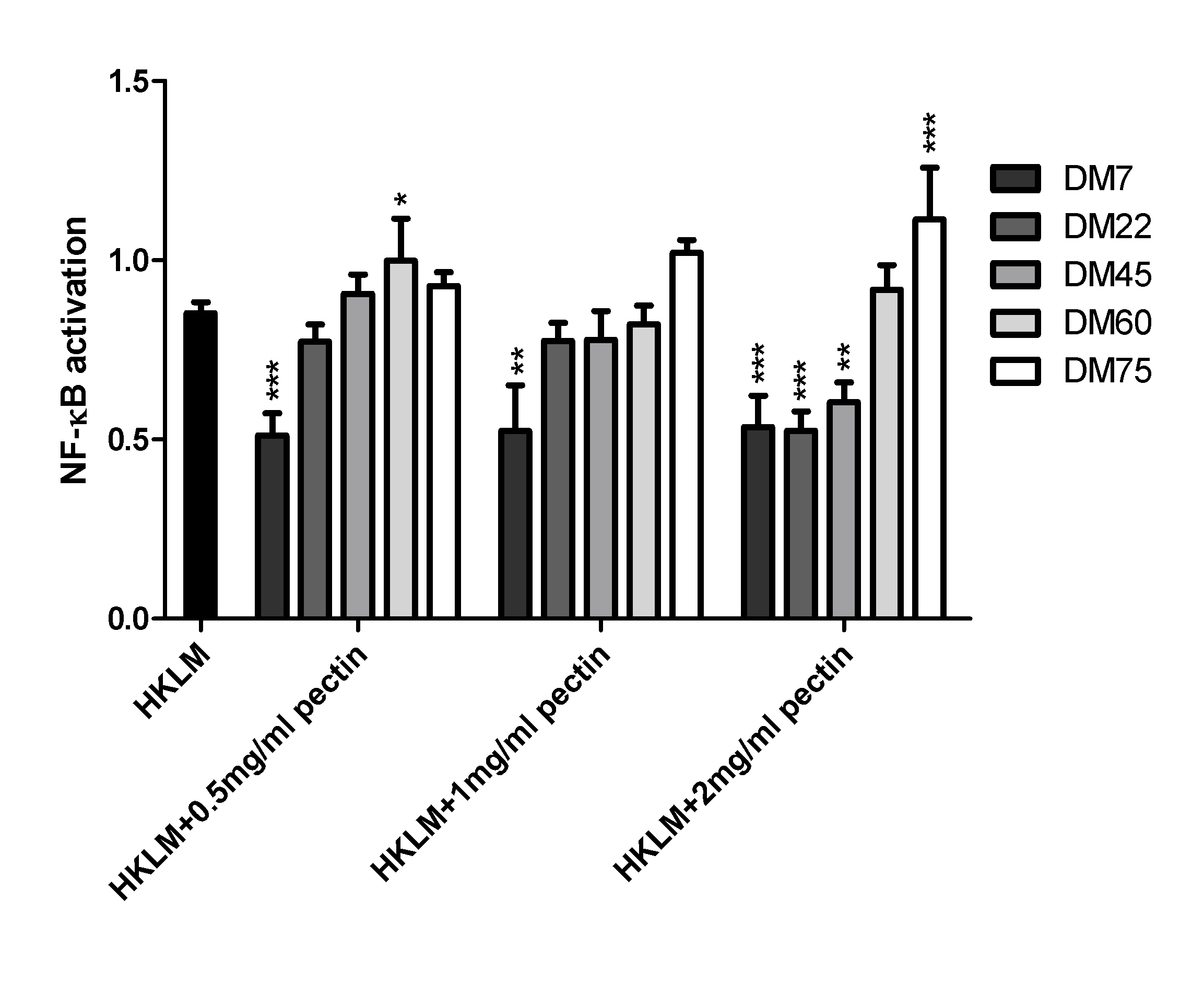

Supplement: Figure S2 — Pectin inhibits Heat killed Listeria monocytogenes (HKLM) stimulated Toll-like Receptor (TLR) 2. TLR inhibition in HEK reporter cell lines for TLR2 which were pretreated with pectin followed by activation by HKLM (n=5). Data are presented as the mean ± SD, and statistical significance was calculated using two-way ANOVA analysis (*p < 0.001). [file image_2.tif]
